# Distinct subgroup of the Ras family member 3 (DIRAS3) expression impairs metastasis and induces autophagy of gastric cancer cells in mice

**DOI:** 10.1007/s00432-018-2708-3

**Published:** 2018-07-24

**Authors:** Jingping Qiu, Xiaoting Li, Yingjian He, Dan Sun, Wenhui Li, Yan Xin

**Affiliations:** 1 Laboratory of Gastrointestinal Onco-Pathology, Cancer Institute and General Surgery Institute, The First Affiliated Hospital of China Medical University, No. 155 Nanjing North Street, Heping District, Shenyang, 110001 China; 2 Key laboratory of Carcinogenesis and Translational Research (Ministry of Education), Peking University Cancer Hospital and Institute, Beijing, 100142 China

**Keywords:** DIRAS3, Autophagy, Metastasis, Gastric cancer

## Abstract

**Purpose:**

Distinct subgroup of the Ras family member 3 (DIRAS3), also called Aplasia Ras homolog member I, is a tumor suppressor gene that induces autophagy in several cancer cell lines.

**Methods:**

This study analyzed DIRAS3, and markers of autophagy (p62, and LC3B-II) in surgically resected GC samples from 420 patients. The promotion of autophagy by DIRAS3 in gastric cancer (GC) cells was explored, which might explain its inhibitory role in gastric cancer cells.

**Results:**

DIRAS3 expression in GC was positively correlated with LC3B-II amount, and negatively with metastasis; DIRAS3 and p62 levels were independent prognostic factors in GC. Overexpression of DIRAS3 in BGC-823 cells induced autophagy, led to decreased proliferation, cell cycle arrest in G0/G1 phase, increased apoptosis, and impaired migration and invasion. While knockdown of DIRAS3 promoted proliferation and migration in MKN-45 cells. Overexpression of DIRAS3 in BGC-823 cells elevated autophagy levels in subcutaneous xenograft and inhibited tumor growth in mice; the hematogenous liver and lung metastasis of cancer cells were also suppressed.

**Conclusions:**

In conclusion, the results suggest DIRAS3 may play a role in affecting proliferation and metastatic potential of GC cells, which may be associated with its involvement in autophagy regulation.

## Background

Basal autophagy eliminates damaged cellular components and the resulting breakdown products are released from lysosomes and recycled into metabolic and biosynthetic pathways (White et al. 2015). But the role of autophagy in cancer is contextual: it may either protect the cancer cells (Lock and Debnath 2008; Lu et al. 2008; Rao et al. 2014) or play an anti-cancer role (Hashimoto et al. 2008; Liu et al. 2013; Saiki et al. 2011). Some cancers use autophagy-mediated recycling to meet their high metabolic demand for growth and proliferation. On the other hand, autophagy prevents the buildup of toxins and so limits oxidative stress, chronic tissue damage, and oncogenic signaling (White et al. 2015). Some researchers, therefore, consider autophagy to impede early cancer development while facilitating advanced tumor progression and metastasis (Kenific 2015).

LC3B-II is a marker for autophagic structures, and its turnover is widely used as an indicator to monitor autophagy flux (Klionsky et al. 2016; Mizushima et al. 2010). p62, an oncogene, acts as a signal hub between autophagy and proteasome degradation pathways (Moscat and Diaz-Meco 2009). p62 accumulates due to the lack of effective degradation in autophagy-deficient conditions, suggesting that the increase in p62 expression may indicate autophagy deficiency (Ichimura et al. 2008).

Distinct subgroup of the Ras family member 3 (DIRAS3), also known as Aplasia Ras homolog member I (ARHI), is a tumor suppressor gene that is involved in tumor development (Yu et al. 1999) and autophagy (Klingauf et al. 2013; Lu et al. 2008). For example, in ovarian and breast cancers DIRAS3 has been found to inhibit cell migration, induce autophagy and increase sensitivity to chemotherapy (Lu and Bast Jr 2013; Lu 2014; Washington et al. 2015; Zou et al. 2011). To our knowledge, only three studies have investigated the effect of DIRAS3 on the biological behaviors of gastric cancer (GC) cells in vitro (Li et al. 2013; Tang et al. 2012; Wang et al. 2012). These previous studies suggest that DIRAS3 expression is negatively correlated with cancer cell survival and that its expression might inhibit proliferation, foci formation, and invasiveness in culture (Li et al. 2013; Tang et al. 2012; Wang et al. 2012).

Therefore, we hypothesized that the role DIRAS3 plays in GC may in part be related to its role in autophagy. So, this study aimed to explore the regulatory mechanisms involving DIRAS3, autophagy and metastasis in GC cells.

## Methods

### Patients

The GC specimens and paired adjacent normal mucosa (more than 5 cm from the lesion margin) were collected from 420 patients undergoing curative resection of their GC at the Department of Oncology Surgery, the First Affiliated Hospital, China Medical University. None of the patients had received radiotherapy or chemotherapy before surgery. All methods were carried out in accordance with relevant guidelines and regulations, and all experimental protocols were approved by the China Medical University. Informed consent was obtained from all subjects for the use of the samples.

### Tissue microarray

The GC and adjacent normal mucosa specimens were fixed in 10% formalin, embedded in paraffin, and cut into 4-µm sections. Twelve blocks of tissue microarray containing GC tissues and adjacent normal mucosa were constructed using a microarrayer (Beecher Instruments, USA), and then cut into 4-µm serial sections and placed on glass slides (Hu et al. 2014).

### Immunohistochemistry

The GC and adjacent normal mucosa specimens were fixed in 10% formalin, embedded in paraffin. Tissue microarray constructed using a microarrayer (Beecher Instruments, USA) (Hu et al. 2014). DIRAS3, LC3B-II and p62 protein amounts were detected using two-step immunohistochemistry (Fuzhou Maixin, DAB-0031; Beijing Zhongshan Goldenbridge Co., LTD, PV-9000), according to the manufacturer’s instructions with the following primary antibodies: rabbit anti-DIRAS3 polyclonal antibody (Sigma), rabbit anti-LC3B polyclonal antibody (cell signaling), and mouse anti-human p62 monoclonal antibody (MBL). The anti-LC3B antibody used to identify LC3B-II also reacts with LC3B-I but in that case the staining is diffuse. Therefore, positive expression of DIRAS3 or LC3B-II was defined as the presence of brown granular or punctate granules in the cytoplasm, and for p62, the positive reaction was limited to the cytoplasm or nucleus. The levels of DIRAS3, and p62 and LC3B-II (punctate staining) were assessed by two blinded observers using two randomly selected high-power fields to count 200 cells and assign a proportion score and an intensity score to cancer cells and gastric mucosal epithelial cells. The proportion score was given according to the proportion of positive cells (0, < 10%; 1, ≥ 10%). The intensity score represented the average intensity of positive cells (0, none; 1, weak and intermediate; 2, strong). The final scores were the products of the proportion and intensity scores, ranging from 0 to 2. The expression was categorized into negative (−, score 0–1) or positive (+, score 2).

### Cell culture and overexpression transfection constructs

A panel of four human GC cell lines and human immortalized gastric epithelial cell line GES-1 were maintained in our laboratory and grown in RPMI1640 (Gibco) supplemented with 10% fetal bovine serum (Gibco). BGC-823 cells were transfected with Lipofectamine 2000 (Invitrogen) for the C-terminally tagged shuttle plasmid pCMV6-DIRAS3-AC-GFP or vector plasmid pCMV6-AC-GFP (Origene). Positive clones were selected repeatedly under a selection stress with 800 ng/µL G418 (Gibco, 11811-023) to establish stably transfection cell lines, with 200 ng/µL of G418 as the maintenance concentration.

### Quantitative real-time reverse transcriptase PCR (qRT-PCR)

Total RNA was isolated using the EASY spin plus RNA kit (Aidlab), mRNA levels were quantified by the SYBR Green qPCR method, and relative expression was calculated using the delta–delta CT method (2^−△△Ct^). Primer sequences were: *DIRAS3* (forward) 5′-CCC GCC CTG CTT ATC CT-3′, (reverse) 5′-CGT CGC CAC TCT TGC TGT-3′; *P62* (forward) 5′-CTG GCG GAG CAG ATG AG-3′, (reverse) 5′-TGG CGG GAG ATG TGG GTA-3′; *GAPDH*: (forward) 5′-GAA GGT GAA GGT CGG AGT C-3′, (reverse) 5′-GAA GAT GGT GAT GGG ATT TC-3′.

### Western blotting

Western blotting was conducted as previously reported (Noman et al. 2011). Primary antibodies (Ab) against DIRAS3 (HPA029384) were purchased from Sigma; p62 (M162-3) was from MBL; LC3B (cst3868), ERK_1/2_ (cst4695), pERK_1/2_ (Thr202/Tyr204, cst4370), AKT (cst4691), pAKT (Ser473, cst4060), SP1 (cst9389), and vimentin (cst5741) were from Cell Signaling Technology; NF-κB (p65) (sc-109), αE-Catenin (cs-1495), MMP-2 (sc-13595), and MMP-9 (sc-21733) were from Santa Cruz; p-mTOR (Ser2448, bs-3494R) was from Bioss; β-Catenin (610154) was from BD, p-STAT3 (Ser727,11046) was from SAB; VEGF (ab46154) was from Abcam; and MIIP (orb100491) was from Biorbyt.

### Epigenetic modifiers 5-AZA-dC and TSA

Cells were treated with the DNA methylation inhibitor 5-AZA-dC (5 and 10 µM) for 5 days, or the histone deacetylase inhibitor TSA (50, 100, 200, and 300 nM) for 1 day before collection.

### Mouse models of subcutaneous xenograft

BALB/c-nu mice were purchased from the Shanghai SLAC Laboratory Animal Co. The 4- to 5-week-old male mice weighing 20–22 g were raised under specific pathogen-free (SPF) conditions at the Animal Experiment Center, China Medical University. We subcutaneously injected 5 × 10^6^/200 µL DIRAS3-BGC-823 cells or vector-BGC-823 cells into the right neck within 30 min (*n* = 5/group). Mice were killed 4 weeks post-injection. The tumor sizes were measured by a Vernier caliper, and the tumor volume was calculated as (*L* × *W*^2^)/2 where *L* is the length and *W* is the width of the tumor.

### Mouse model of hematogenous metastasis

To verify the role of DIRAS3 in metastasis in vivo, we used a nude mice model of hematogenous metastasis with lung and liver metastasis initiated via tail vein injection. Briefly, 5 × 10^6^/100 µL DIRAS3-BGC-823 cells or vector-BGC-823 cells were injected into the tail vein for each of two groups (*n* = 5/group). Mice were killed after anesthesia by intraperitoneal injection of pentobarbital 8 weeks post-injection. Lungs and liver were removed, fixed in 4% neutral formalin, and embedded in paraffin (Ogino et al. 2011).

### Statistical analysis

Data were analyzed using SPSS 17.0. All data from qRT-PCR, MTT, clonogenic assay, flow cytometric analysis, wound healing assay, transwell assay and epigenetic modifiers assay were obtained from at least three independent experiments and expressed as the mean ± standard deviation. Statistical analyses of the results were performed with one-way ANOVA. The relationship between clinicopathological factors, immunohistochemistry experiments, protein level, and GC were evaluated with the chi-square test. Protein level on survival was evaluated with the Kaplan–Meier method, using the log-rank test. *P* < 0.05 was considered statistically significant.

## Results

### DIRAS3, LC3B-II and p62 protein amounts are associated with the clinicopathological characteristics in gastric cancer

**Fig. 1:**
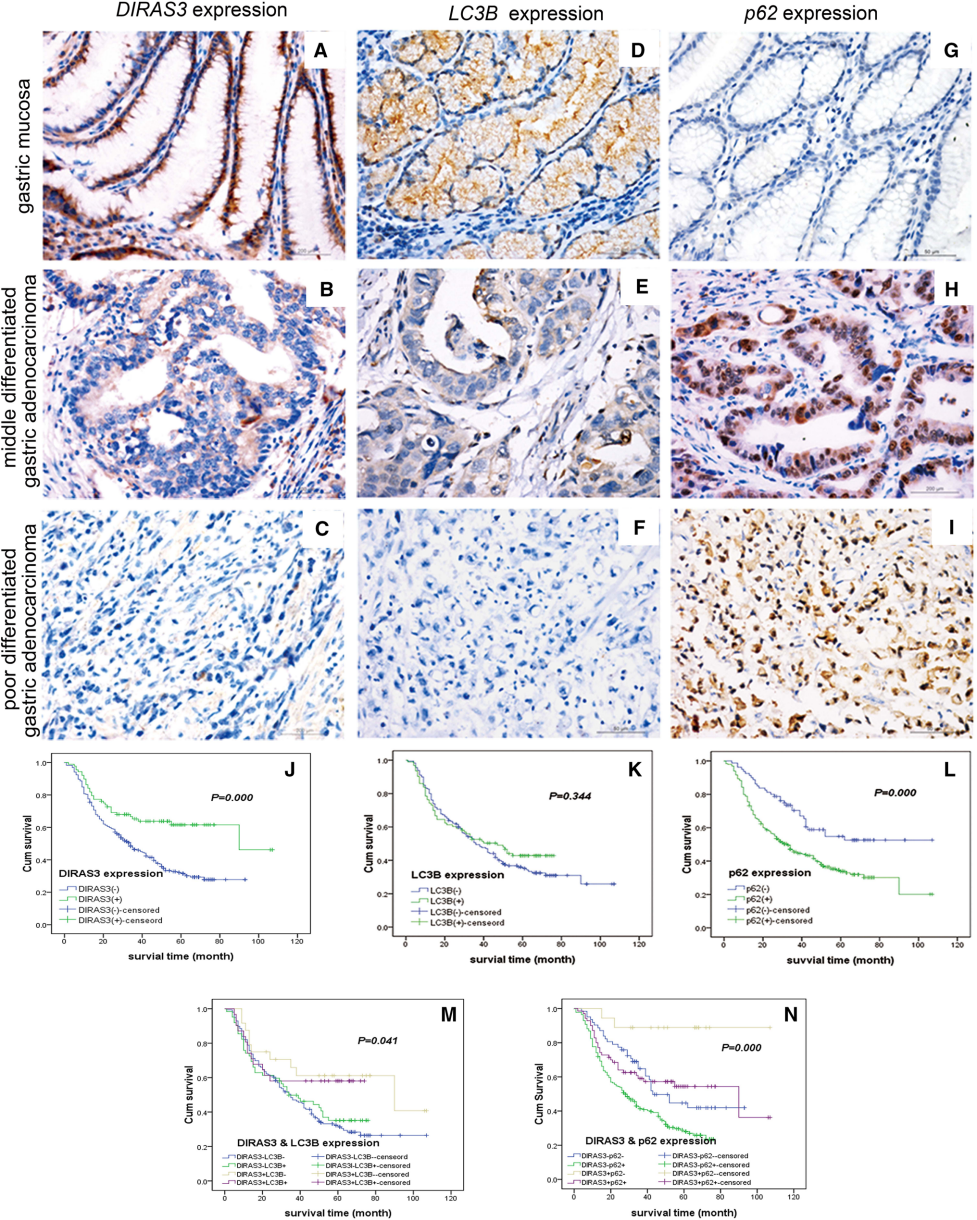
Representative images of immunostaining of DIRAS3, LC3B-II and p62 in gastric adenocarcinoma and adjacent normal gastric mucosa specimens; and clinical significance of DIRAS3, LC3B-II and p62 amount for gastric cancer patients. **a, d, g** In the adjacent normal gastric mucosa, DIRAS3 and LC3B-II were positive, and p62 was negative. **b, e, h** In moderately differentiated gastric adenocarcinoma, the amount of DIRAS3 and LC3B-II was reduced, while p62 was accumulated. **c, f, i** In poorly differentiated gastric carcinoma with signet ring cell carcinoma, DIRAS3 and LC3B-II were negative, and p62 was strongly positive. For **a**–**i**, magnification: ×400. **j**–**l** Kaplan–Meier curves depicting the overall survival and showing that DIRAS3^+^ implied a better prognosis than DIRAS3^−^, p62^−^ implied a better prognosis than p62^+^, and LC3B-II was not involved in prognosis. **m, n** The interaction analysis of DIRAS3 with LC3B-II and of DIRAS3 with p62 showed that the worst prognosis was observed in the DIRAS3^−^LC3B-II^−^ group, a better prognosis was observed in the DIRAS3^−^LC3B-II^+^ group, a slightly worse prognosis was observed in the DIRAS3^+^LC3B-II^+^ group and the best prognosis was in the DIRAS3^+^LC3B-II^−^ group

Preliminary study reported an association between DIRAS3 expression and clinicopathological features in a small sample of 81 pT2 stage gastric cancer specimens, but without association with autophagy. So, we used LC3B-II and p62 levels as markers to detect autophagy (Klionsky et al. 2016) and investigated the levels of DIRAS3, p62 and LC3B-II (indicated by punctate staining), and their clinical significance in 420 gastric cancer specimens. Using immunohistochemistry, we determined the levels of DIRAS3, p62 and LC3B-II in gastric cancer and adjacent normal gastric mucosa. The specific immunostaining of DIRAS3 and LC3B-II occurred in the cytoplasm, as granular/punctate staining, while it occurred in the cytoplasm and nuclei for p62 (Fig. 1a–i). The percentage of cells positive for DIRAS3 and LC3B-II were significantly lower in gastric cancer than in adjacent mucosa (DIRAS3, 24 vs. 74%, LC3B-II 31 vs. 87%, both *P* < 0.05), whereas the percentage of cells positive for p62 was significantly higher in gastric cancer than in adjacent mucosa (79 vs. 57%, *P* < 0.05) (Table 1).

**Table 1:** Correlations of *DIRAS3, p62* and *LC3B* expressions with the clinicopathological parameters in gastric cancer

Variable	DIRAS3 expression (*n* = 420)					p62 expression (*n* = 420)					LC3B expression (*n* = 304)				
	Positive	Negative	PR (%)	*χ* ^2^	*P*	Positive	Negative	PR (%)	*χ* ^2^	*P*	Positive	Negative	PR (%)	*χ* ^2^	*P*
Pathological diagnosis				212.102	**0.000**				48.228	**0.000**				191.734	**0.000**
Adjacent mucosa	311	109	74.05			238	182	56.67			263	41	86.51		
Gastric cancer	100	320	23.81			332	88	79.05			95	209	31.25		
Age (year)				0.421	0.516				1.944	0.163				3.224	0.073
≪ 55	33	117	22.00			113	37	75.33			42	70	37.5		
> 55	67	203	24.81			219	51	81.11			53	139	27.6		
Gender				1.291	0.256				0.282	0.596				0.019	0.891
Female	25	99	20.16			96	28	77.42			28	60	31.82		
Male	75	221	25.34			236	60	79.73			67	149	31.02		
Borrmann’s types				0.001	0.976				16.850	**0.000**				0.066	0.798
I + II	13	43	23.21			33	23	58.93			15	31	32.61		
III + IV	84	275	23.40			297	62	82.73			78	176	30.71		
WHO’s histology types					0.081*					0.746*					0.067*
Papillary. Ade.	6	5	54.55			8	3	72.73			4	1	80.00		
Tubular Ade.															
Well Diff.	7	13	35.00			15	5	75.00			4	14	22.22		
Moderately Diff.	43	118	26.71			130	31	80.75			37	79	31.90		
Poorly Diff.	34	144	19.10			140	38	78.65			39	88	30.71		
Un-differentiated car	0	4	0.00			2	2	50.00			0	4	0		
Signet ring cell car	1	7	12.50			7	1	87.50			0	7	0		
Mucinous Ade.	9	29	23.68			30	8	78.95			11	16	40.74		
Lauren’s types				6.165	**0.046**				1.496	0.473				5.099	0.078
Intestinal	45	102	30.61			118	29	80.27			27	65	29.35		
Diffuse	43	179	19.37			177	45	79.73			46	117	28.22		
Mixed	12	39	23.53			37	14	72.55			22	27	44.90		
Depth of invasion					0.270*					0.031*					0.025*
T1	3	3	50.00			2	4	33.33			2	2	50.00		
T2	14	45	23.73			45	14	76.27			19	25	43.18		
T3	50	144	25.77			151	43	77.84			58	118	32.95		
T4	33	128	20.50			134	27	83.23			16	64	20		
Ln metastasis				9.038	**0.003**				7.018	**0.008**				0.079	0.779
N0	38	73	34.23			78	33	70.27			26	54	32.5		
N1–3	62	247	20.06			254	55	82.20			69	155	30.8		
Distant metastasis				4.583	**0.032**				11.642	**0.001**				3.15	0.076
M0	79	217	26.69			221	75	74.66			62	157	28.31		
M1	21	103	16.94			111	13	89.52			33	52	38.82		
TNM staging				11.729	**0.008**				12.854	**0.005**				6.936	0.074
I	13	21	38.24			23	11	67.65			11	15	42.31		
II	34	73	31.78			80	27	74.77			25	57	30.49		
III	32	123	20.65			118	37	76.13			26	85	23.42		
IV	21	103	16.94			111	13	89.52			33	52	38.82		
LC3B expression				12.966	**0.000** ^**a**^				0.000	0.985					
Negative	34	175	16.27			163	46	77.99							
Positive	33	62	34.74			74	21	77.89							
p62 expression				0.302	0.583										
Negative	19	69	21.59												
Positive	81	251	24.40												

PR% in each row was calculated as: the number of positive cases/(the number of positive cases + the number of negative cases) × 100%

PR, positive rate; Histol., histological; Papi., papillary; ade., adenocarcinoma; diff., differentiation; Undiff., undifferentiated; Car, carcinoma; SRC, signet ring cell cancer; Ln, lymph node

*Fisher exact test

^a^ *r* = 0.207

To identify the clinical significance of level of DIRAS3 and the level of autophagy, we examined the associations between DIRAS3, p62 and LC3B-II levels with the clinicopathological characteristics of gastric cancer. The level of DIRAS3 protein was associated with Lauren’s type, lymph node metastasis, distant metastasis, and TNM stage, but not with age, gender, Borrmann’s type, WHO’s histological type, and depth of invasion. The level of DIRAS3 in patients with stage N1 to N3 was at a significantly lower rate than that in patients with stage N0 (*P* < 0.001, Table 1). In patients with metastasis, DIRAS3 levels in the primary lesion were significantly lower than in patients without metastasis. The level of p62 protein was associated with Borrmann’s type, depth of invasion, lymph node metastasis, distant metastasis and TNM stage, but not with age, gender, WHO’s histological type, and Lauren’s type. The level of LC3B-II was only associated with the depth of invasion (Table 1). Besides, DIRAS3 level in gastric cancer was significantly positively associated with LC3B-II level, and further correlation analysis showed a weak correlation between level of DIRAS3 and LC3B-II (*r* = 0.21, Table 1), but no correlation was found between the level of DIRAS3 and p62 or LC3B-II and p62 protein level (Table 1).

In this study, 378 patients had available follow-up data. Among them, 220 died of gastric cancer, with a median survival of 39 months (range 1–107 months) and overall survival rate of 41.8%. Kaplan–Meier survival analysis and log-rank test were performed to evaluate the effects of DIRAS3, p62 and LC3B-II protein levels on survival. The overall survival rate was significantly higher in the DIRAS3^+^ group than in DIRAS3^−^ group, and it was significantly higher in the p62^−^ group than in p62^+^ group (Fig. 1j–l). Multivariate Cox regression model showed that the expressions of DIRAS3 (HR = 0.576, 95% CI 0.369–0.899, *P* = 0.02) and p62 (HR = 1.695, 95% CI 1.134–2.534, *P* = 0.01) were independent prognosis factors, but LC3B-II was not (Table 2).

**Table 2:** Univariate and multivariate analysis of different prognostic factors in 378 patients with gastric cancer

Variable	Univariate analysis^a^			Multivariate analysis^b^	
	*n*	Mean survival (months, 95% CI)	*P* value	HR (95% CI)	*P* value
Lauren’s types			**0.020**	1.105 (0.799–1.290)	0.901
Intestinal	133	61.02 (53.02–69.01)			
Diffuse	197	45.76 (39.69–51.83)			
Mixed	48	44.96 (36.25–53.66)			
Depth of invasion			**0.000**	2.324 (1.301–4.149)	**0.004**
T1 + T2	58	78.88 (68.09–89.67)			
T3 + T4	320	48.07 (43.14–53.01)			
Ln metastasis			**0.000**	2.272 (1.481–3.485)	**0.000**
N0	102	75.76 (67.13–84.38)			
N1–3	276	43.87 (38.61–49.12)			
Distant metastasis			**0.000**		
M0	256	71.22 (65.58–76.85)			
M1	122	13.87 (11.99–15.75)			
TNM staging			**0.000**		
I	29	95.25 (84.60-105.89)			
II	98	83.12 (75.59–90.65)			
III	129	55.59 (48.00-63.18)			
IV	122	13.87 (11.99–15.75)			
*DIRAS3* expression (*n* = 378)			**0.000**	0.576 (0.369–0.899)	**0.015**
Negative	290	44.19 (40.06–48.32)			
Positive	88	70.41 (60.42–80.40)			
*p62* expression (*n* = 378)			**0.000**	1.695 (1.134–2.534)	**0.010**
Negative	80	70.29 (60.85–79.72)			
Positive	298	47.36 (41.91–52.81)			
*LC3B* expression (*n* = 273)			0.344		
Negative	180	50.29 (44.20-56.37)			
Positive	93	43.94 (37.79–50.09)			
*DIRAS3*&*p62* expression			**0.000**	1.013 (0.726–1.413)	0.940
DIRAS3^+^ p62^−^	18	97.17 (84.31-110.03)			
DIRAS3^+^ p62^+^	70	63.63 (52.31–74.95)			
DIRAS3^−^ p62^−^	62	56.49 (47.62–65.36)			
DIRAS3^−^ p62^+^	228	36.75 (33.12–40.37)			
*DIRAS3*&*LC3B* expression			**0.041**		
DIRAS3^+^ LC3B^−^	24	69.36 (51.90-86.82)			
DIRAS3^+^ LC3B^+^	31	48.23 (37.47–58.99)			
DIRAS3^−^ LC3B^−^	156	47.94 (41.63–54.25)			
DIRAS3^−^ LC3B^+^	62	41.17 (33.91–48.43)			

Ade, adenocarcinoma; Diff, differentiated; car, carcinoma; Ln, lymph node

^a^Log rank test

^b^Cox regression model

To evaluate the role of autophagy regulation of DIRAS3 in prognosis, we tested the interaction of DIRAS3 and LC3B-II, and the interaction of DIRAS3 and p62 (Fig. 1m, n). The patients were divided into four groups based on the levels of DIRAS3 and LC3B-II in their primary lesions; and analysis of their survival showed that the worst prognosis was observed in the DIRAS3^−^LC3B-II^−^ group, a better prognosis was observed in the DIRAS3^−^LC3B-II^+^ group, and a much better prognosis was observed in the DIRAS3^+^LC3B-II^+^ group, suggesting that DIRAS3 level affects the prognosis in a stronger way than LC3B-II level. The best prognosis was in the DIRAS3^+^LC3B-II^−^ group. The patients were divided into four groups based on the levels of DIRAS3 and p62 in their primary lesions, and analysis of their survival showed that the worst prognosis was in the DIRAS3^−^p62^+^ group, while the best was in the DIRAS3^+^p62^−^ group, suggesting that the combined detection of DIRAS3 and p62 could improve the predictive effectiveness of gastric cancer prognosis (Table 2).

### BGC-823 showed the lowest expression of DIRAS3 together with the strongest metastatic abilities among GC cell lines

**Fig. 2:**
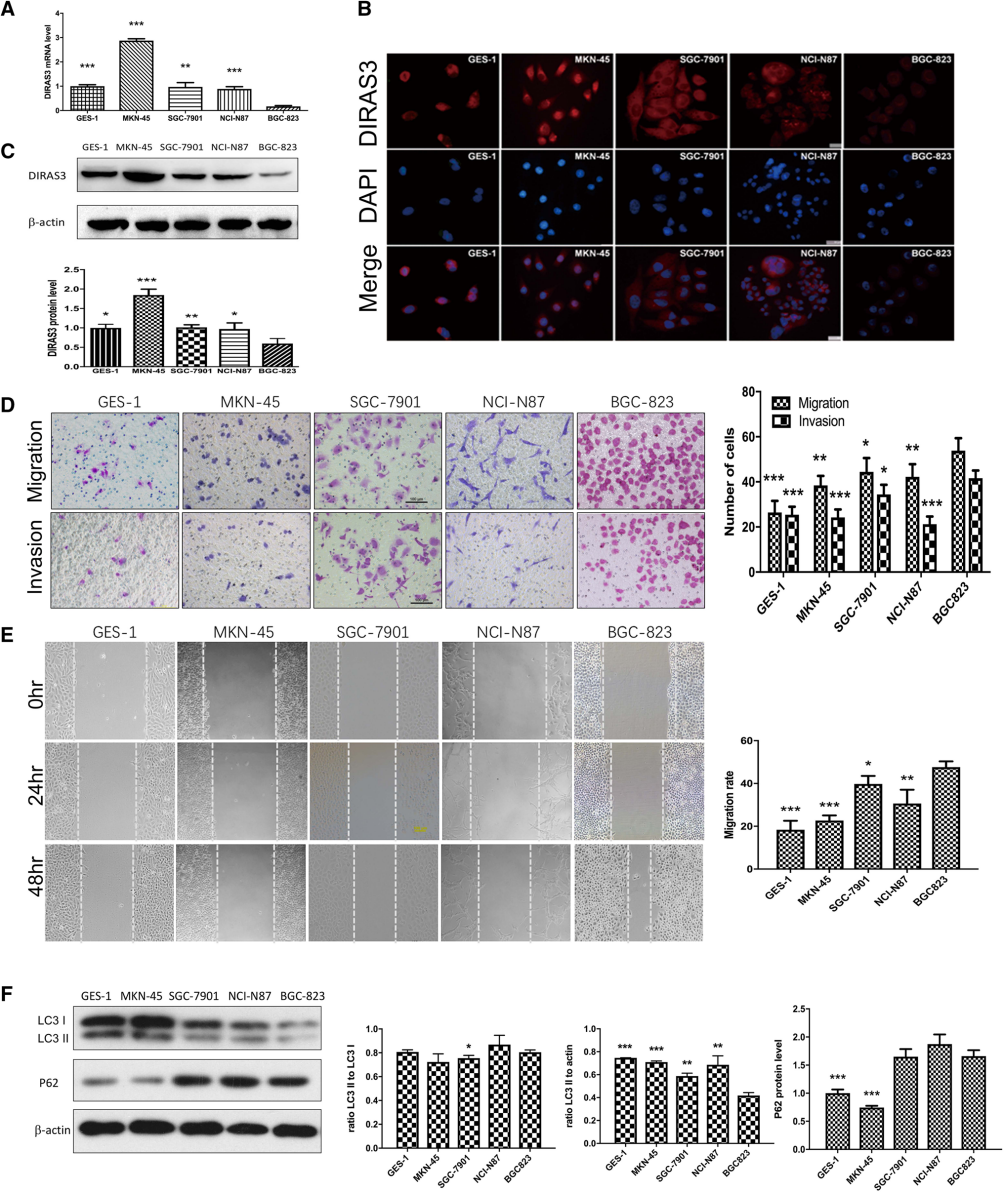
Biologic features of gastric epithelial cell line GES-1 and gastric cancer cell lines MKN-45, SGC-7901, NCI-N87 and BGC-823. **a** The relative level of *DIRAS3* mRNA (normalized to *GAPDH*) detected by qRT-PCR (*n* = 3) in GES-1 (1.00 ± 0.06), MKN-45 (2.88 ± 0.08), SGC-7901 (0.97 ± 0.17), NCI-N87 (0.89 ± 0.09) and BGC-823 cells (0.17 ± 0.04). **b** Immunostaining of DIRAS3 (red) in the five cell lines, with nuclear stained by DAPI (blue). Magnification: for NCI-N87 cells, ×200; for other cells, ×400. **c** The relative DIRAS3 protein level (normalized to β-actin) detected by western blot analysis (*n* = 3) in GES-1 (1.00 ± 0.09), MKN-45 (1.85 ± 0.15), SGC-7901 (1.03 ± 0.07), NCI-N87 (0.97 ± 0.16) and BGC-823 cells (0.60 ± 0.13). **d** Transwell migration and invasion assays (*n* = 5) show the number of migration/invasion cell of GES-1 (26.40 ± 5.18/24.50 ± 3.42), MKN-45 (38.40 ± 4.22/24.20 ± 3.56), SGC-7901 (44.40 ± 6.19/34.40 ± 4.28), NCI-N87 (42.20 ± 5.63/21.20 ± 3.42) and BGC-823 cells (53.80 ± 5.54/41.6 ± 3.44). Magnification for crystal violet staining: ×100. **e** Wound healing assay (*n* = 3) shows migration rate (48 h) of GES-1 (18.38 ± 4.22), MKN-45 (22.69 ± 2.39), SGC-7901 (39.78 ± 3.76), NCI-N87 (30.62 ± 6.44) and BGC-823 cells (47.62 ± 2.72). Scale bar 100 µm. **f** Western blot analysis (*n* = 3) shows relative LC3B-II amount (normalized to β-actin) in GES-1 (0.75 ± 0.00), MKN-45 (0.71 ± 0.01), SGC-7901 (0.59 ± 0.03), NCI-N87 (0.69 ± 0.08) and BGC-823 cells (0.42 ± 0.03); the ratio of LC3B-II to LC3B-I in GES-1 (0.81 ± 0.02), MKN-45 (0.72 ± 0.07), SGC-7901 (0.75 ± 0.02), NCI-N87 (0.87 ± 0.08) and BGC-823 (0.81 ± 0.02) cells; the relative P62 protein level (normalized toβ-actin) in GES-1 (1.00 ± 0.06), MKN-45 (0.75 ± 0.03), SGC-7901 (1.65 ± 0.14), NCI-N87 (1.87 ± 0.17) and BGC-823 cells (1.66 ± 0.11). **P < 0.05, **P < 0.01 and ***P < 0.001 vs. BGC-823 cells

The expression *DIRAS3* of was evaluated in gastric epithelial cell line GES-1 and a panel of four gastric cancer cell lines: MKN-45, SGC-7901, NCI-N87 and BGC-823. The qRT-PCR, immunofluorescence and western blot showed *DIRAS3* was observed in all cell lines tested, with the lowest level being in BGC-823 cells (Fig. 2a–c). The immunofluorescence showed that the positive staining of DIRAS3 was mainly in the cytoplasm. On the other hand, we compared the metastatic capacities among the gastric cancer cell lines. The results showed that BGC-823 had strongest migratory and invasive abilities (Fig. 2d, e).

To investigate the expression of DIRAS3 in BGC-823 cells hindered by transcriptional regulation such as DNA methylation and histone deacetylation, we found treating BGC-823 cells with a DNA methylation inhibitor, 5-aza-2′-deoxycytidine (5-AZA-dC) for 5 days, or with a histone deacetylase inhibitor, trichostatin A (TSA) for 1 day resulted in increases in the levels of *DIRAS3* mRNA, respectively (Supplementary Fig. 1). These results suggested that promoter methylation and histone acetylation could be important causes of down-regulation of DIRAS3 in BGC-823 cells.

### DIRAS3 overexpression inhibits proliferation, migration and invasion of BGC-823 cells possibly associated with promoting autophagy

**Fig. 3:**
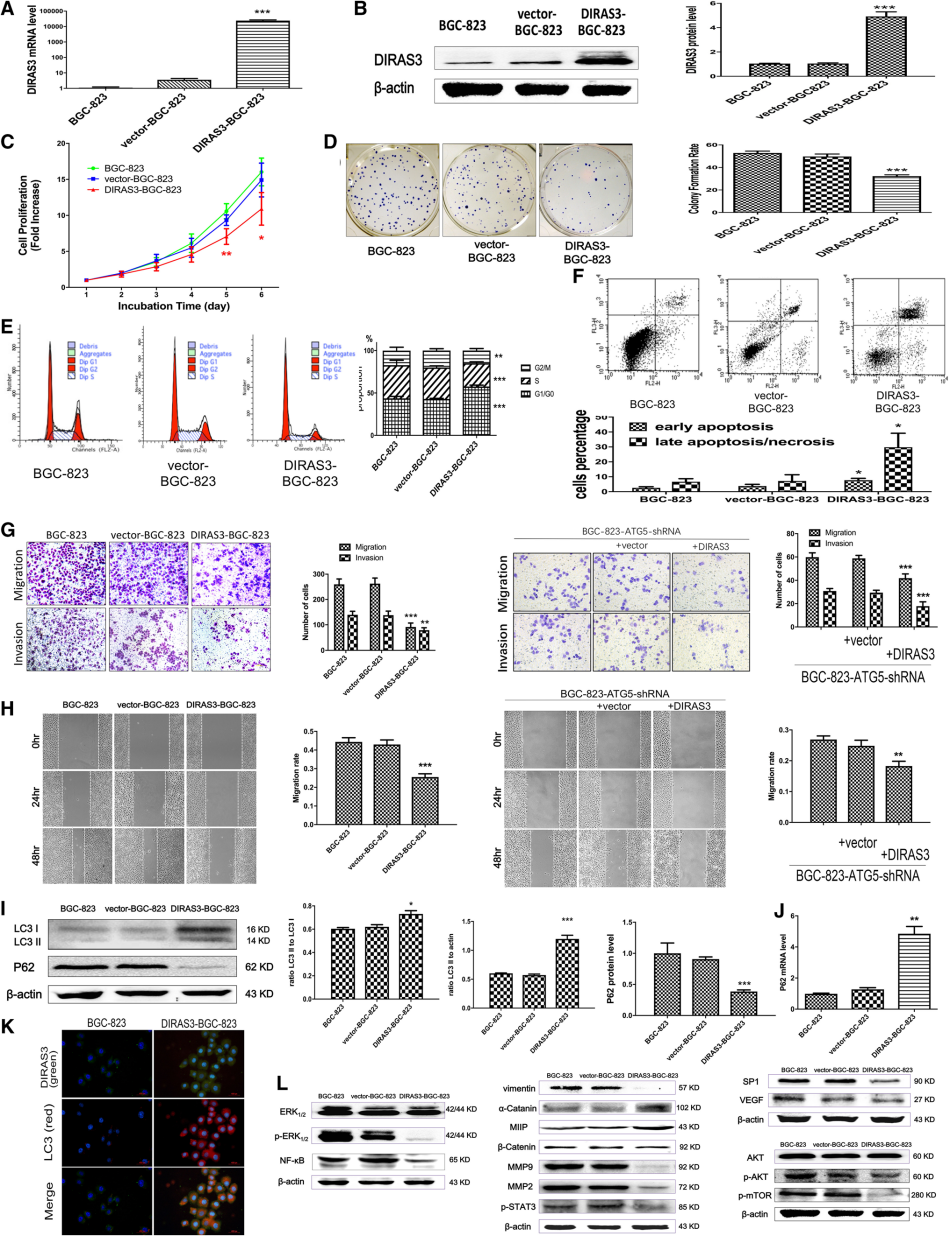
Biologic features of overexpression of DIRAS3 in gastric cancer cell line BGC-823. **a** The relative level of *DIRAS3* mRNA (normalized to *GAPDH*) detected by qRT-PCR (*n* = 3) in BGC-823, vector-BGC-823 and DIRAS-BGC-823. **b** The relative DIRAS3 protein level (normalized to β-actin) detected by western blot analysis (*n* = 3). **c** Cell proliferation rate was measured with MTT colorimetric assay (*n* = 5) in BGC-823 (10.58 ± 1.05 on Day 5, 16.03 ± 1.93 on Day 6), vector-BGC-823 (9.33 ± 0.74 on Day 5, 14.92 ± 2.36 on Day 6) and DIRAS3-BGC-823 cells (7.06 ± 1.08 on Day 5, 10.90 ± 2.27 on Day 6). **d** The colony formation assay (*n* = 3) shows the colony formation rate of BGC-823 (49.70 ± 2.20%), vector-BGC-823 (52.80 ± 1.70%) and DIRAS3-BGC-823 cells (32.30 ± 1.20%). **e** Flow cytometry (*n* = 5) shows the proportions of cells in G0/G1 phase for BGC-823 (43.68 ± 23.20%), vector-BGC-823 (42.95 ± 0.70%) and DIRAS3-BGC-823 cells (57.60 ± 1.66%); in S phase 39.17 ± 5.75, 37.12 ± 1.92, 27.52 ± 1.61%, respectively; in G2/M phase 17.15 ± 3.99, 19.94 ± 2.21, 14.89 ± 2.40%, respectively. **f** Apoptosis analysis with flow cytometry (*n* = 3) in BGC-823 (early apoptosis: 2.59 ± 0.74%; late apoptosis: 6.65 ± 2.05%), vector-BGC-823 (early: 3.76 ± 1.19%; late: 7.17 ± 4.18%) and DIRAS3-BGC-823 cells (early: 7.69 ± 1.24%; late: 29.78 ± 9.78%). **g** Transwell migration and invasion assays (*n* = 3) show the number of migration/invasion cells for BGC-823 (259.67 ± 21.01/139.67 ± 13.58) and vector-BGC-823 (263.33 ± 22.03/138.00 ± 16.09) and DIRAS3-BGC-823 cells (91.33 ± 15.63/78.67 ± 9.61). Transwell assays (*n* = 5) also show the number of migration/invasion cell of BGC-823-ATG5 shRNA (59.80 ± 3.77/30.80 ± 2.17), BGC-823-ATG5 shRNA-vector (58.80 ± 2.59/29.40 ± 2.07) and BGC-823-ATG5 shRNA-DIRAS3 (41.80 ± 3.70/17.80 ± 3.77). Magnification for crystal violet staining: ×100. **h** Wound healing assay (*n* = 5) shows migration rate (48 h) is 44.36 ± 2.24, 42.98 ± 2.41 and 25.51 ± 1.76 for BGC-823, vector-BGC-823 and DIRAS3-BGC-823. Wound healing assay (*n* = 3) also shows migration rate (48 h) of BGC-823-ATG5 shRNA (26.89 ± 1.16), BGC-823-ATG5 shRNA-vector (24.84 ± 1.81) and BGC-823-ATG5 shRNA-DIRAS3 cells (18.28 ± 1.56). **i** Western blot analysis (*n* = 3) shows relative LC3B-II amount (normalized to β-actin) is 0.60 ± 0.01, 0.57 ± 0.03 and 1.20 ± 0.11 for BGC-823, vector-BGC-823 and DIRAS3-BGC-823 cells; the ratio of LC3B-II to LC3B-I is 0.60 ± 0.02, 0.62 ± 0.03 and 0.73 ± 0.05 for BGC-823, vector-BGC-823 and DIRAS3-BGC-823 cells; the relative P62 protein level (normalized toβ-actin) is 1.00 ± 0.29, 0.91 ± 0.06 and 0.39 ± 0.05 for BGC-823, vector-BGC-823 and DIRAS3-BGC-823 cells. **j** The level of *p62* mRNA detected by qRT-PCR (*n* = 3) is 0.99 ± 0.04, 1.27 ± 0.11 and 4.85 ± 0.47 for BGC-823, vector-BGC-823 and DIRAS3-BGC-823 cells. **k** Immunochemical staining of LC3B in DIRAS3-BGC-823 and BGC-823 cells. Punctate staining of LC3 indicated LC3B-II. **l** Western blot analysis of several signaling pathways in BGC-823, vector-BGC-823 and DIRAS3-BGC-823 cells, with β-Actin as the internal control. *P < 0.05, **P < 0.01 and ***P < 0.001 vs. vector-BGC-823 cells

We then choose BGC-823 cells to ascertain whether the aggressiveness of these gastric cancer cells would be suppressed by DIRAS3 overexpression. The effectiveness of overexpression was verified by qRT-PCR and western blotting (Fig. 3a, b, Supplementary Fig. 2). To investigate the effects of DIRAS3 overexpression in BGC-823 cells, we evaluated the cell proliferation, migration, invasion as well as autophagy level in BGC-823, vector-BGC-823 and DIRAS3-BGC-823 cells.

First, methyl thiazolyltetrazolium (MTT) assay (Fig. 3c) and clonogenic assay (Fig. 3d) showed DIRAS3 overexpression led to reduced proliferation rates and colony formation rate in BGC-823 cells, suggesting that DIRAS3 suppressed clonogenicity of gastric cancer cells and thus inhibited proliferation. The flow cytometry showed the proportions of cells in G0/G1 phase increased, and the proportion of cells in S phase and G2/M phase decreased in DIRAS3-BGC-823 cells (Fig. 3e). The results revealed the proportion of early and late apoptotic DIRAS3-BGC-823 cells were significantly increased (Fig. 3f), suggesting DIRAS3 overexpression induced early and late apoptosis in BGC-823 cells, which may be considered as another cause of impaired cell proliferation in addition to the G0/G1 phases cycle arrest.

Next, to examine the role of DIRAS3 on migration and invasion in BGC-823 cells, transwell migration/invasion assay and wound healing assay were performed. Both the transwell migration and invasion assays showed that the numbers of cells entering the lower chamber were significantly reduced in DIRAS3-BGC-823 cells compared with BGC-823 and vector-BGC-823 cells (Fig. 3g). And the wound healing assay also showed that the wound healing rate of the DIRAS3-BGC-823 cells was significantly decreased compared with BGC-823 and vector-BGC-823 cells (Fig. 3h). Thus, these results suggested that DIRAS3 overexpression could reduce the migration and invasion ability of BGC-823 cells to some extent.

To explore the role of DIRAS3 on the autophagy, the level of LC3B-II, the LC3B-II/I ratio and the level of p62 were evaluated. BGC-823 had a relatively lower level of autophagy among several gastric cancer cell lines, as indicated by the lowest level of LC3B-II (normalized to β-actin) as well as the relatively higher level of p62 (Fig. 2f). Compared with BGC-823 and vector-BGC-823 cells, the level of LC3B-II and the LC3B-II/I ratio was increased, and the amount of p62 protein was decreased in DIRAS3-BGC-823 cells, and the punctate staining of LC3B-II increased suggesting that DIRAS3 overexpression may increase the level of autophagy in BGC-823 cells (Fig. 3i, k). On the contrary, the level of *p62* mRNA was significantly increased in DIRAS3-BGC-823 cells compared with vector-BGC-823 cells (Fig. 3j), which may result from the compensatory adjustment for promoted degradation of p62 protein.

Furthermore, to test whether DIRAS3-induced gastric cancer cell migration depends upon autophagy, we successfully knocked down autophagy-initiating factor ATG5 together with DIRAS3 overexpression in BGC-823 cells (Supplementary Fig. 3). We compared the migration rates in BGC-823, BGC-823-control shRNA and BGC-823-ATG5 shRNA cells by scratch healing experiments (Supplementary Fig. 4). The migration of BGC-823-ATG5 shRNA increase compared with BGC-823-con shRNA, suggesting that the knockdown of ATG5 in BGC-823 cells increases the migration of BGC-823 cells (Supplementary Fig. 4). In spite of ATG5 knockdown, the overexpression of DIRAS3 still impaired migration (Fig. 3g, h). These results indicated that DIRAS3 might affect the metastatic capacity of gastric cancer cells by means other than autophagy.

### DIRAS3 knockdown increases proliferation and migration in MKN-45 cells

**Fig. 4:**
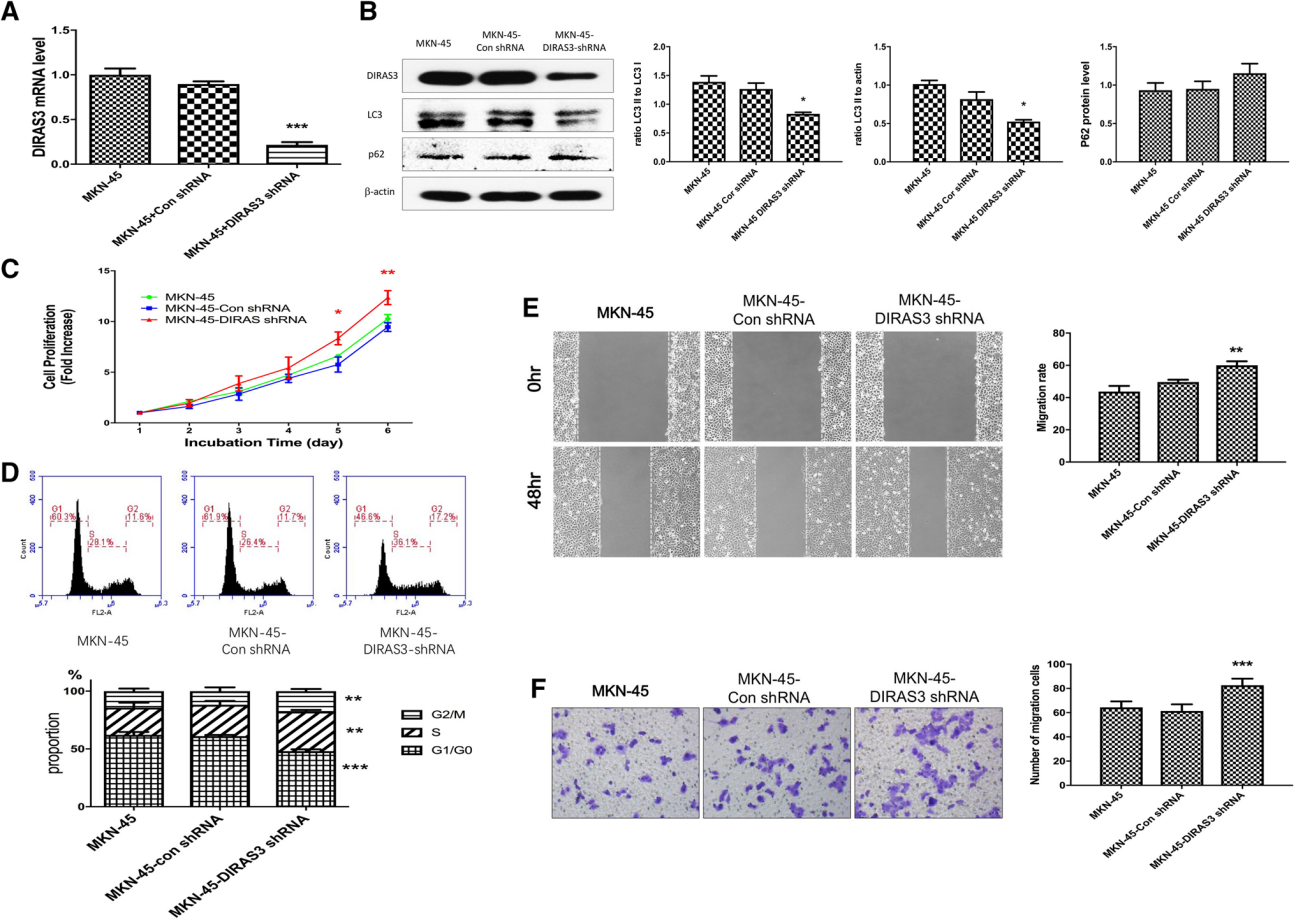
Biologic features of knockdown of DIRAS3 in gastric cancer cell line MKN-45. **a** The relative level of *DIRAS3* mRNA detected by RT-PCR (*n* = 3) in MKN-45, MKN-45-Con shRNA and MKN-45-DIRAS-shRNA cells. **b** The relative protein levels of DIRAS3, LC3B and p62 detected by western blot analysis (*n* = 3). **c** Cell proliferation rate was measured with MTT colorimetric assay (*n* = 3) in MKN-45 (6.61 ± 0.11 on Day 5, 10.27 ± 0.43 on Day 6), MKN-45-Con shRNA (5.75 ± 0.75 on Day 5, 9.46 ± 0.44 on Day 6) and MKN-45-DIRAS-shRNA cells (8.34 ± 0.63 on Day 5, 12.35 ± 0.69 on Day 6). **d** Flow cytometry (*n* = 5) shows the proportions of cells in G0/G1 phase for MKN-45 (62.07 ± 2.47%), MKN-45-Con shRNA (60.97 ± 1.02%) and MKN-45-DIRAS-shRNA cells (47.98 ± 1.53%); in S phase 23.68 ± 4.33, 27.28 ± 3.29, 34.35 ± 1.39%, respectively; in G2/M phase 14.25 ± 2.24, 11.75 ± 3.30, 17.67 ± 1.93% respectively. **e** Wound healing assay (*n* = 3) shows migration rate (48 h) is 43.68 ± 3.56, 49.63 ± 1.46 and 60.00 ± 2.48 in MKN-45, MKN-45-Con shRNA and MKN-45-DIRAS-shRNA cells. **f** Transwell migration assays (*n* = 5) show the number of migration cells for MKN-45 (64.49 ± 4.88) and MKN-45-Con shRNA (61.40 ± 5.50) and MKN-45-DIRAS-shRNA cells (82.60 ± 5.46). Magnification for crystal violet staining: ×100. *P < 0.05, **P < 0.01 and ***P < 0.001 vs. MKN-45-Con shRNA cells

To verify the role of DIRAS3 in proliferation and migration, DIRAS3 knockdown was successfully constructed in MKN-45 cells (Fig. 4a, b) and the autophagy level, the cell proliferation and migration were then evaluated by western blot, MTT, transwell migration assay and wound healing assay in MKN-45, MKN-45-Con shRNA and MKN-45-DIRAS3-shRNA cells. The overall level of LC3B-II and the ratio of LC3B-II to LC3BI were both decreased in MKN-45-DIRAS3-shRNA cells and p62 showed an increasing trend, though this was not significant, suggesting that silencing of DIRAS3 may decrease the level of autophagy in MKN-45 cells. Silencing of DIRAS3 caused an increase of cell proliferation in MKN-45 cells by MTT assay. Next, the cell cycle was assessed and the results showed a decreased proportion at G0/G1 cells and increased number of cells at S and G2/M phases in MKN-45-DIRAS3-shRNA cells. These results indicated DIRAS3 down-regulation promotes cell proliferation by accelerating cell cycle progression in MKN-45 cells. In addition, wound healing assay showed an increased migration rate and the transwell migration assay showed an increased number of migration cells in MKN-45-DIRAS3-shRNA cells. These data indicated the DIRAS3 down-regulation was capable of promoting cell proliferation and migration in MKN-45 cells.

### DIRAS3 overexpression regulates the biological behavior of BGC-823 cells via multiple signaling pathways

Western blot analyses of several signaling pathways were performed (Fig. 3l). This showed that in DIRAS3-BGC-823 cells, the levels of p-ERK1/2 and NF-κB were decreased, while the levels of ERK1/2 protein remained unchanged. These results suggest that DIRAS3 overexpression may inhibit the cell proliferation by inhibiting the p-ERK1/2 pathway, and may induce cell apoptosis by inhibiting the NF-κB signal pathway. We also found decreased levels of vimentin, MMP2, MMP9, and p-STAT3 proteins, and increased levels of α-catenin and MIIP (migration and invasion inhibitory protein) proteins. No differences in β-catenin protein were observed between DIRAS3-BGC-823 cells and BGC-823 or vector-BGC-823 cells. We speculated that DIRAS3 overexpression could reverse the epithelial–mesenchymal transition and thereby inhibit migration and invasion of BGC-823 cells. In addition, we found that SP1 and vascular endothelial growth factors (VEGF) were decreased in DIRAS3-BGC-823 cells, suggesting that DIRAS3 expression may inhibit tumor growth by inhibiting angiogenesis. We further found that the total levels of AKT protein remained unchanged, p-AKT (Ser473) was decreased, and p-mTOR was decreased in DIRAS3-BGC-823 cells compared with the control groups. Based on these results, we speculated that DIRAS3 overexpression inhibits the malignant behavior of gastric cancer cells possibly by reducing the activity of the p-AKT/mTOR pathway.

### DIRAS3 overexpression inhibits the growth of subcutaneous tumors

**Fig. 5:**
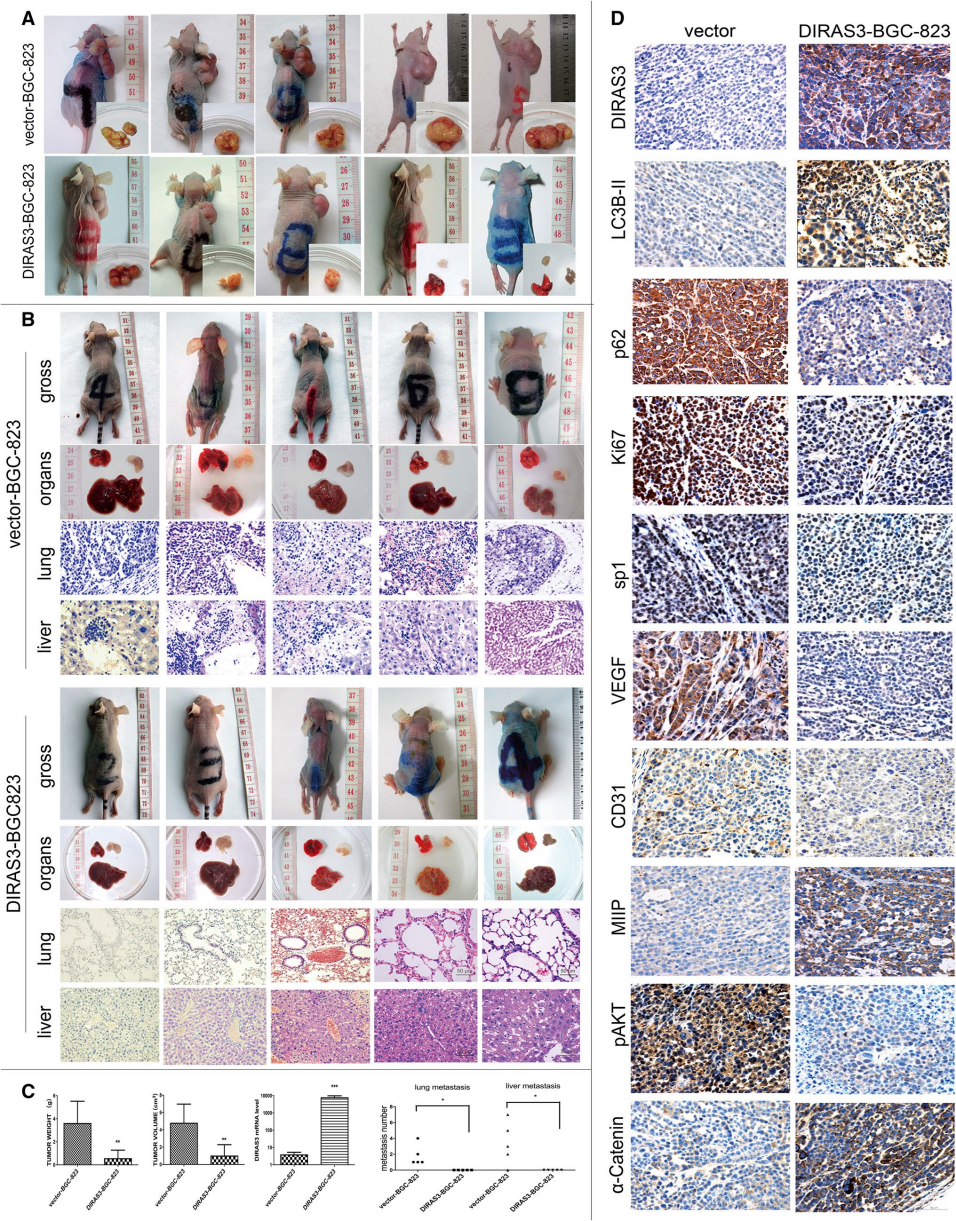
Subcutaneous xenograft models and hematogenous metastasis model in BALB/c-nu mice. **a** The BALB/c-nu mice and subcutaneous xenograft models. The volume of the xenograft formed from DIRAS3-BGC-823 cells was less than that from vector-BGC-823 cells. **b** The hematogenous metastasis in BALB/c-nu mice xenograft model. In the vector-BGC-823 group, lung metastatic foci were found by naked eyes in three mice, decentralized lung metastatic foci were found in two mice, and liver metastatic foci were found in four mice. No liver metastasis was found in one mouse (H&E staining; magnification: ×400); in the DIRAS3-BGC-823 group, no lung or liver metastasis was found in all five mice after careful observation by microscope (H&E staining; magnification: ×200). **c** Compared with the vector-BGC-823 group, the volume and weight of subcutaneous xenograft was reduced in the DIRAS3-BGC-823 group, and the *DIRAS3* mRNA levels of subcutaneous xenograft was higher, as detected by qRT-PCR. The number of lung and liver metastatic foci was higher in the hematogenous metastasis model. **d** Compared with the vector-BGC-823 group, the subcutaneous xenograft of the DIRAS3-BGC-823 group showed higher levels of DIRAS3 and LC3B-II, lower levels of p62, and the Ki67-positive cell proportion was higher. The expressions of SP1 and VEGF were down-regulated, and CD31-positive microvessel number was lower in the DIRAS3-BGC-823 group. The levels of MIIP and α-catenin were higher and the levels of p-AKT were lower (immunohistochemistry; magnification: ×400). *P < 0.05, **P < 0.01 and ***P < 0.001 vs. vector-BGC-823 group

Given the in vitro observation that DIRAS3 overexpression impaired the metastatic capacity of BGC-823 cells, we further established a mice model of subcutaneous xenograft to verify the effect in vivo. The results of qRT-PCR (Fig. 5c) and immunohistochemistry (Fig. 5d) showed the mRNA and protein levels of *DIRAS3* in the subcutaneous xenograft maintained higher in DIRAS3-BGC-823 group than vector-BGC-823, indicating the effectiveness of the mice model.

We compared the growth of the subcutaneous xenograft. The subcutaneous tumors developed 4 weeks after the inoculation of gastric cancer cells (Fig. 5a, b), with the tumor formation rate of 100% in both vector-BGC-823 and DIRAS3-BGC-823 groups. Compared with the vector-BGC-823 group (Fig. 5c), the tumor weight was significantly lower (3.63 ± 1.90 vs. 0.58 ± 0.70 g, *P* < 0.01) and the tumor volume was significantly smaller (4.83 ± 2.15 vs. 1.03 ± 1.25 cm^3^, *P* < 0.01) in the DIRAS3-BGC-823 group, indicating that DIRAS3 overexpression could inhibit the growth of subcutaneous tumor formed from BGC-823 gastric cancer cells in vivo.

Next, we verified the levels of autophagy in subcutaneous tumors by immunohistochemistry of p62 and LC3B-II (Fig. 5d), and the results showed the level of LC3B-II (indicated by the punctate staining of LC3B as the diffuse staining was LC3B-I) was significantly increased, while the expression of p62 was significantly reduced, suggesting that DIRAS3 overexpression may further inhibit subcutaneous tumor growth by inducing autophagy, compared with the vector-BGC-823 group.

In addition, to further elucidate the involvement of DIRAS3 with the proliferation, angiogenesis, migration and invasions, we performed immunohistochemistry of Ki67, SP1, VEGF, CD31, MIIP, pAKT, and α-catenin in subcutaneous xenograft (Fig. 5d). In terms of granule distribution, positive results were found in the nucleus for Ki67 and SP1, in the cytoplasm for VEGF, MIIP, pAKT, and α-catenin, and in vascular endothelial cells for CD31. Ki67 is a marker of cell proliferation. The percentage of Ki67-positive cells in DIRAS3-BGC-823 xenograft examined by IHC staining was lower in the DIRAS3-BGC-823 xenograft than the vector-BGC-823 xenograft, indicating DIRAS3 overexpression inhibited the proliferation of xenograft cells, therefore, inhibited the growth of the subcutaneous xenograft. CD31 is a marker of microvessels, and SP1 and VEGF are essentials of the regulative pathway. The IHC showed that microvessels were fewer and the expressions of SP1 and VEGF were down-regulated in DIRAS3-BGC-823 xenograft compared with the vector-BGC-823 xenograft, suggesting that DIRAS3 overexpression inhibited angiogenesis of xenograft through the SP1/VEGF signal pathway. MIIP was reported as a novel indicator of migration and invasion inhibition (Ji et al. 2010). Compared with vector-BGC-823 xenograft, the expression of MIIP was remarkably up-regulated in DIRAS3-BGC-823 xenograft, indicating that DIRAS3 overexpression could inhibit migration and invasion by up-regulating the expression of MIIP. In addition, p-AKT and α-catenin are extensively involved in the proliferation, migration and invasion. The IHC showed down-regulation of p-AKT and up-regulation of α-catenin in DIRAS3-BGC-823 xenograft, possibly indicating that DIRAS3 expression inhibited the malignant behavior of gastric cancer cells by the p-AKT and α-catenin pathways.

### DIRAS3 inhibits lung and liver metastases in nude mice

A mice model of hematogenous metastasis by vein injection with the DIRAS3-BGC-823 or vector-BGC-823 tumor cells. Each group was assigned five mice, the mice in both groups lost weight with a reduction of subcutaneous fat, while food intake and activity were decreased concomitantly from the fifth week after injection. The mice were killed 8 weeks post-injection, and no pleural effusion or ascites was found in either group. The lung and liver tissues were paraffin embedded and stained with hematoxylin and eosin. The results showed that DIRAS3 significantly reduced the number of metastatic foci (Fig. 4b). A lower number of liver and lung metastatic foci were found in the mice in the DIRAS3-BGC-823 group (*n* = 5, 0/5 mice, 0 nodules per mouse), compared with lung metastatic nodules (*n* = 5, 5/5 mice, 1.8 ± 1.3 nodules per mouse) and liver metastatic nodules (*n* = 5, 4/5 mice, 3.4 ± 2.7 nodules per mouse) in vector-BGC-823 group (Fig. 5c) (*P* = 0.037 for lung metastasis and *P* = 0.048 for liver metastasis). The data suggest that DIRAS3 expression decreases the possibility of hematogenous metastasis to liver and lung of BGC-823 cells.

## Discussion

Low expression of DIRAS3 is associated with high malignancy of ovarian, breast, and prostatic cancers, while high expression predicts good prognosis of ovarian and pancreatic cancers (Dalai et al. 2007). A similar study undertaken previously showed that DIRAS3 expression in GC is down-regulated compared with normal gastric mucosa, and a high expression of DIRAS3 indicated a high survival rate (Wang et al. 2012). So, it seems that DIRAS3 expression is negatively correlated with GC cell survival and that its expression might inhibit proliferation, foci formation, and invasiveness in culture (Tang et al. 2012; Wang et al. 2012). However, the relationship between DIRAS3 expression in clinical specimens and metastasis is still unclear, and as far as we know there have been no studies relating to DIRAS3 and its role in autophagy in GC. This study found that DIRAS3, as a tumor suppressor gene, is an independent prognostic factor in GC and that this may be related to increased levels of autophagy when DIRAS3 is expressed.

LC3B-II, indicated by a punctate pattern of distribution as opposed to the diffuse staining of LC3B-I, is often considered as a marker for autophagic structures (Klionsky et al. 2016). LC3 was increased in 53% of esophageal cancer, 58% of GC, and 63% of colorectal cancer (Yoshioka et al. 2008), while LC3 in hypopharyngeal cancer and renal clear cell cancer was decreased (Wang et al. 2013). High expression of LC3 in oral squamous cell cancer, esophageal cancer, and melanoma indicated a poor prognosis (Tang et al. 2013), while low expression of LC3 was associated with poor prognosis in hypopharyngeal cancer and renal clear cell cancer (Wang et al. 2013). P62 is considered as an indicator of autophagy flux (Mizushima et al. 2010). High expression of p62 was related to poor prognosis in oral squamous cell cancer and triple-negative breast cancer, but the expression of p62 and LC3 were not associated with prognosis in colon and breast cancers (Luo et al. 2012). In the present study concerning gastric cancer, LC3B-II amount was not associated with prognosis while p62 amount was an independent prognosis factor (Table 2). It should be noted that it is the turnover of LC3B-II rather than the amount of LC3B-II, which could in fact indicate the autophagy flux (Mizushima et al. 2010). The different significances of LC3B-II amount and its turnover in monitoring authentic autophagy might help to explain this discrepancy. It was suggested that the level of LC3B-I should also be detected so as to provide a full picture of the cellular autophagic response apart from measuring LC3B-II amount relative to home-keeping proteins (Klionsky et al. 2016). Hence in this study, the quantification of LC3B-II (normalized to β-actin) and the calculation of LC3B-II/LC3B-I ratio were both presented. In this study, the turnover of LC3 II has not been examined using lysosome inhibitor in the in vitro experiments. However, the amount of p62 was examined as another indicator of autophagy flux (Mizushima et al. 2010).

Previous observations indicate that the expression of DIRAS3 induces autophagy in ovarian cancer cells at several steps, including participating directly in the initiation complex and activating the nuclear localization of autophagy-related transcription factor FOXO3 to permit fusion of autophagosomes with lysosomes (Lu et al. 2014a, b). In the present study, we found an association between DIRAS3 expression and LC3B-II amount, but no correlation between DIRAS3 and p62 expressions in GC tissues was observed, suggesting that DIRAS3 might participate in the induction of autophagy in GC but it might not be as important as other factors during the progression of autophagy.

In our study, to test whether DIRAS3-induced gastric cancer cell migration depends upon autophagy, we chose to establish a stable knockdown of ATG5 in BGC-823 gastric cancer cells. In macroautophagy, autophagosomes are formed by closure of cup-shaped isolation membranes. The Atg12–Atg5 conjugate system plays essential roles in isolation membrane development and is essential for autophagy (Mizushima et al. 2001). Hence, specific inhibition of the autophagy pathway can be achieved by knockout or knockdown of Atg5 (Mizushima et al. 2010). In the ATG5-knockdown, we think that the shRNA itself may affect cell viability, so the loss of cell migration caused by ATG5 knockdown may be a non-specific effect of shRNA. Migration of BGC-823-control shRNA significantly decreased compared with BGC-823, and the migration of BGC-823-ATG5 shRNA increases compared with BGC-823-con shRNA, suggesting that the knockdown of ATG5 in BGC-823 cells increases the migration of BGC-823 cells. Further overexpression of DIRAS3 still inhibited migration after ATG5 knockdown. Therefore, we speculated that the effect of DIRAS3 on cell migration was achieved by mechanisms including but not limited to autophagy.

This study showed that DIRAS3 overexpression inhibits the formation of metastatic foci in lung and liver at the proliferation/anti-apoptosis steps, angiogenesis, motility, intravasation, and survival in the vasculature. Earlier studies have reported DIRAS3, as a member of the Ras superfamily of small G proteins, to be an anti-oncogene expressed in breast, pancreatic, and ovarian cancers (Hu et al. 2013). DIRAS3 overexpression can induce cell cycle arrest in G0/G1 and promote cell cycle arrest in G2/M induced by the HDAC (histone deacetylase) inhibitor TAS in ovarian and breast cancers (Zou et al. 2011).

During the angiogenesis step, we found that DIRAS3 overexpression inhibited the SP1/VEGF pathway. DIRAS3 overexpression reduced the phosphorylation of S6K1 and 4E-BP1, two mTOR substrates, and the levels of HIF-1 and VEGF (Zhao et al. 2010). Vascular endothelial growth factor (VEGF) and its receptors are central regulators of angiogenesis, tumor growth, and metastasis in various tumors (Lohela et al. 2009). Sp1 promotes the metastasis of colon adenocarcinoma cells (Takami et al. 2007). Our study showed that DIRAS3 suppressed the expression of VEGF by inhibiting SP1. MMP2 and MMP9 are related to metastasis and angiogenesis in tumors (Huang et al. 2002). Our results also indicated that DIRAS3-expression could suppress the expressions of MMP2 and MMP9.

We also found that DIRAS3 overexpression inhibited extracellular matrix degradation, inhibited cell migration and invasion, reversed epithelial–mesenchymal transition (EMT), and repressed the p-ERK pathway, which are associated with lower aggressiveness (Ding et al. 2015). MIIP can inhibit the formation and invasion of glioma cells, induce mitotic catastrophe, and inhibit cell migration and invasion in breast cancer (Ji et al. 2010). Our study revealed that DIRAS3 overexpression inhibits tumor migration and invasion by up-regulating MIIP in GC cells. Previous studies have found that in a model of chemotactic migration, DIRAS3 forms a complex with STAT3 or p-STAT3 in the cytoplasm to prevent STAT3 from translocating to the nucleus and binding to DNA (Huang et al. 2010). In the haptotaxic migration model, DIRAS3 expression decreased the expression of β1 integrin and inhibited the phosphorylation of FAK (Lu and Bast Jr 2013). A study showed that DIRAS3 was induced to accumulate at the cell membrane and bind to C-RAF to specifically suppress the phosphorylation of MEK and ERK and thus inhibit cell migration (Klingauf et al. 2013). Our study also showed that DIRAS3 overexpression decreased the levels of p-ERK, indicating that the suppression of the p-ERK pathway inhibited migration in GC cells.

Last, we observed that DIRAS3 overexpression induced markers of autophagy and autophagic cell death by inhibiting the PI3K–AKT–mTOR pathway. An earlier study demonstrated overexpression of DIRAS3 in ovarian cancer cells inhibited both basal and lysophosphatidic acid-induced activation of AKT (Lu et al. 2008). Further results showed DIRAS3 inhibits PI3K activity and membrane localization of AKT. DIRAS3 inhibits the PI3K/AKT and Ras/ERK signaling pathway by enhancing internalization and degradation of the epidermal growth factor receptor (Lu et al. 2014a). However, PI3K–AKT–mTOR signaling compensated by tumor microenvironment could rescue the ovarian cancer cells from autophagic death and the metastatic tumor fell into dormancy in vivo (Lu et al. 2008).

Down-regulation of DIRAS3 is achieved through several mechanisms, including loss of heterozygosity, DNA methylation, transcriptional down-regulation by E2F transcription factor 1 (E2F1) and E2F transcription factor 4 (E2F4), shortened RNA half-life and inhibition by microRNAs (Lu et al. 2014a; Sutton et al. 2018). DNA demethylation agents and/or histone deacetylation inhibitors can recover DIRAS3 activity in breast and ovarian cancers (Badgwell et al. 2011). Suppressing methylation of DIRAS3 by Zebularine can elevate DIRAS3 expression and enhances apoptosis in osteosarcoma cells (Ye et al. 2016). In addition, DNA over-methylation occurs in 79.1% of GC tissue with deficient DIRAS3 expression (Wang et al. 2012). In the present study, DNA methylation transferase inhibitor 5-AZA-dC and histone deacetylase inhibitor TSA up-regulated the expression of *DIRAS3* mRNA. Thus, epigenetics may be a novel strategy of GC treatment through overexpression of DIRAS3.

This study has some limitations. The small sample size means that statistical significance may have been missed in some of the subgroups and that there is a slight contradiction in the results showing the prognosis of the subgroups. While the increase in LC3B-II amount combined with the reduction in p62 levels suggests the induction of autophagy, and more methods for monitoring autophagosome number and autophagic flux would improve the understanding of relationship of autophagy and migration. We were unable to perform electron microscopy to identify autophagosomes; this would be an important approach in future.

In conclusion, this study suggests that DIRAS3 may play a role in affecting proliferation and metastatic potential of GC cells, which may be associated with its involvement in autophagy regulation.

## Abbreviations

ARHI: Aplasia Ras homolog member I
GC: Gastric cancer
DIRAS3: Distinct subgroup of the Ras family member 3
qRT-PCR: Quantitative real-time reverse transcriptase PCR
MTT: Methyl thiazolyltetrazolium assay
SPF: Specific pathogen-free
TSA: Trichostatin A
VEGF: Vascular endothelial growth factors
